# Antimalarial Activities of Hydromethanolic Crude Extract and Chloroform Fraction of *Gardenia ternifolia* Leaves in *Plasmodium berghei* Infected Mice

**DOI:** 10.1155/2020/6674002

**Published:** 2020-12-29

**Authors:** Tezera Jemere Aragaw, Dessie Tegegne Afework, Kefyalew Ayalew Getahun

**Affiliations:** ^1^Department of Pharmacology, College of Medicine and Health Sciences, University of Gondar, Gondar, Ethiopia; ^2^Department of Medical Laboratory, College of Health Sciences, Debre Tabor University, Debre Tabor, Ethiopia

## Abstract

**Background:**

*Gardenia ternifolia* is utilized in traditional medicine of Ethiopia for malaria treatment and possessing in vitro antimalarial activity. However, no in vivo study was conducted to substantiate the claim. The aim of this study was to judge the antimalarial activity of *Gardenia ternifolia* extract in vivo in *Plasmodium berghei*-infected mice.

**Methods:**

*Plasmodium berghei* was inoculated to healthy mice, and hydromethanolic crude extract and chloroform fraction of *G. ternifolia* leaves at 100 mg/kg/day, 200 mg/kg/day, and 400 mg/kg/day were administered. Percent parasitemia inhibition, percent change in bodyweight, hemoglobin level, and mean survival time were determined. Data were analyzed using one-way ANOVA followed by post hoc Tukey HSD test with IBM SPSS software version 20.0 statistical package and *P* < 0.05 considered as statistically significant.

**Results:**

The chemosuppressive test of hydromethanolic crude extract at 100 mg/kg/day, 200 mg/kg/day, and 400 mg/kg/day ranged from 27.09% to 67.72%, and chloroform fraction had 35.21%–78.19% parasitemia suppression, respectively. For curative test on day 5, hydromethanolic crude extract at 100 mg/kg/day, 200 mg/kg/day, and 400 mg/kg/day ranged from 25.58% to 48.76%, chloroform fraction at 100 mg/kg/day, 200 mg/kg/day, and 400 mg/kg/day and chloroquine base at 10 mg/kg showed 46.36%–74.42% and 92.87% percent parasitemia inhibition, respectively, and also the results to both tests were highly significant (*P* < 0.001) compared to the negative control. Maximum effects on chemosuppressive, curative, prevention of weight loss, and reduction in hemoglobin were observed at higher doses of the hydromethanolic crude extract and chloroform fraction.

**Conclusion:**

From this study, hydromethanolic crude extract and chloroform fraction of *G. ternifolia* leaves have shown promising antimalarial activity. The findings support the traditional claim of *G. ternifolia* leaves for malaria treatment; however, species variation could also limit such a straightforward extrapolation of the findings of this study in humans.

## 1. Background

Globally, in 2019, there were an estimated 229 million malaria cases of which an estimated 94% (215 million) cases occurred in Africa [1]. Resistance of *Plasmodium falciparum* to the cheap and safe chloroquine (CQ) and to sulfadoxine-pyrimethamine (SP) caused a major drawback in effective malaria control in sub-Saharan Africa [2, 3]. *Gardenia ternifolia* is an evergreen small tree that grows to six meters tall [4]. The intertwined branches have short twigs which are very hard and thorny [5]. Ethnomedical information indicates that *G. ternifolia* (Rubiaceae) has been utilized by traditional healers to palliate malaria and its related fevers [6, 7]. Fresh leaves and edible fruit extracts that are accustomed in managing hemorrhoid lesions showed an antidiabetic activity [8]. The macerated root extract is employed as a laxative and vermifuge for the treatment of stomach aches and kwashiorkor [9].

The crude acetone extract of *G. ternifolia* showed IC_50_ values of 1.06 and 0.94 *μ*g/ml against chloroquine-resistant Indochina (W2) and chloroquine sensitive to Sierra-Leone (D6) strains of *Plasmodium falciparum* [10].

## 2. Rationale of the Study

*Gardenia ternifolia* leaves in vitro and ethnobotanical studies showed antimalarial activity; no report is offered in the literature whether the leaves possess in vivo activity. This study was therefore planned to evaluate the efficacy as well as acute oral toxicity of *G. ternifolia* leaves used as malaria treatment in the Ethiopian folk medicine.

## 3. Materials and Methods

### 3.1. Plant Material

Fresh leaves of *G. ternifolia* were collected on October 13, 2016, from Gondar Zuria district, Degola Chinchaye kebele, about 45 km south of the town, northwest Ethiopia. The collected material was wrapped and covered with plastic sheets during transportation. The specimen of the plant was authenticated as *Gardenia ternifolia* by a botanist (Getnet Chekole), in the Department of Biology, the University of Gondar, and was deposited in the herbarium with a voucher specimen (No. KA1784) for future reference.

### 3.2. Preparations of Hydromethanolic Crude Extract

The leaves of *G. ternifolia* were dried under shade, grounded to a course powder using hand compression, and stored in glass jars at room temperature until extraction. 400 g of *G. ternifolia* leaves powder was macerated in 2400 ml in 80% hydromethanol and shook frequently. After 72 hours of maceration, extraction was performed using thick layers of 40 mesh gauze and filtered with Whatman paper No. 1 twice, and the 2^nd^ extraction was repeated after 72 hours of after maceration; the 3^rd^ extraction was repeated 72 hours later (in a complete of nine days). The extracts were combined and concentrated in a dry oven at 40°C [11].

### 3.3. Preparation of Chloroform Fraction

The hydromethanolic crude extract was subjected to fractionation using solvents with differing polarity (chloroform and water). 40 g of hydromethanolic crude extract powder was suspended in a separatory funnel using 400 ml water and similar volume of chloroform was added and shaken to mix and left for 12 hours. The chloroform portion was powered in a clean beaker and concentrated in a dry oven at 40°C and stored in a refrigerator at 4°C until use [11, 12].

### 3.4. Phytochemical Screening

The qualitative phytochemical screening tests were performed on 80% hydromethanolic crude extract of *G. ternifolia* leaves using standard chemicals and procedures which were utilized in previous researches [13–21].

### 3.5. Animals and Parasite

Swiss albino mice, weighing within 20−28 g, 6–8 weeks old, and either sex, were purchased from the Ethiopian Public Health Institute (EPHI), Addis Ababa. Mice were kept in cages and housed in a standard animal house under a natural 12/12 h light-dark cycle at room temperature with free access to food and water. Before the experiment, they were acclimatized to the test environment for five days. The care and handling were consistent with the international guidelines for the employment and maintenance of experimental animals [22, 23].

Chloroquine-sensitive malaria, *P. berghei*, was obtained from EPHI in the Traditional Medicine Research Department, Addis Ababa, and transported by infecting the donor mice and maintaining the parasite until the actual procedure of the study.

### 3.6. Acute Toxicity Test

Five female mice were kept for five days before dosing to acclimatize. One mouse was randomly taken to work out preliminary toxicity observation on free access to water but fasted for four hours and weighed and hydromethanolic crude extract of *G. ternifolia* leaves at 2000 mg/kg was administered by the oral route. Strict observation was carried out for half an hour and periodically for the first 24 hours, for consecutive fourteen days. Observation was majorly focused on physical and behavioral changes of the mouse. But preliminary toxicity observation of the mouse did not show any sign of toxicity for four days. The remaining 4 mice were dosed by similar doses and followed for 14 days [21, 24].

### 3.7. Pharmacologic Screening (4-Day Suppressive Test)

Donor *P. berghei* infected mice (parasitemia of roughly 30%) were sacrificed by decapitation; then, blood was collected through cardiac puncture with a sterile disposable needle and syringe. The blood was diluted with sterile sodium chloride 0.9% in water in such a way that 0.2 ml of blood contained about 10^7^ infected red blood cells (RBCs) and mice were infected by 0.2 ml blood suspension intraperitoneally expected to produce steadily rising consistent infection of the specified intensity in mice [23]. Then, mice were randomly divided into eight groups of six each, weighed, and maintained on a standard diet. The doses of hydromethanolic crude extract and chloroform fraction were adjusted from the safest dose of acute toxicity study and were 5%, 10%, and 20% from lower to higher doses, respectively. Three different doses got to the mice of hydromethanolic crude extract and chloroform fraction once daily for 4 days. The seventh group was treated with chloroquine base at 10 mg/kg/day (positive control), and the eighth group received 10 ml/kg/day of solvent which contained 3 ml of 96% ethanol + 7 ml of Tween 80 + 90 ml of water (negative control) [25, 26] administered orally using oral gavage, starting from 3 hours of parasite inoculation.

### 3.8. Rane's (Curative) Test

An evaluation of the curative potential of hydromethanolic crude extract and chloroform fraction of *G. ternifolia* leaves was distributed consistent with the strategy described by curative methods [24]. On Day 0, standard inocula of about 10^7^ infected erythrocytes were inoculated in mice intraperitoneally and randomly divided into their respective groups, weighed, and maintained on a pellet diet. Seventy-two hours later, they were dosed with hydromethanolic crude extract at 100 mg/kg/day, 200 mg/kg/day, and 400 mg/kg/day and chloroform fraction at 100 mg/kg/day, 200 mg/kg/day, and 400 mg/kg/day, chloroquine base at 10 mg/kg/day and solvent 10 ml/kg/day, which was prepared from 3 ml of 96% ethanol + 7 ml of Tween 80 + 90 ml of water once daily for five days. A Giemsa-stained thin blood film was prepared from the tail of each mouse daily for five days (days 3, 4, 5, 6, and 7) to determine the parasitemia level. The mean survival time for each group was resolute arithmetically by calculating the mean survival time (days) of mice starting from the date of infection over a period of 30 days (D0-D29) [26].

## 4. Determination of Parasitemia

On day 4 (96 hours), after infection, a drop of blood was collected from the mice by the vein section of the tail and transferred onto the edge of a microscopic slide and drawn evenly across the second slide to create a thin blood film and allowed to dry at room temperature, fixed with methanol, and stained with 10% Giemsa stain for quarter an hour. Slides were viewed using light microscopy with oil immersion (1000 x magnification). Percentage parasitemia was determined by counting the number of parasitized red blood cells (PRBCs) and non-infected red blood cell in random fields of the microscope. Five fields were counted and the parasitemia was calculated as the percentage of the total RBCs containing PRBCs [27].

### 4.1. Percent Parasitemia

Percent parasitemia was calculated according to the following formula [27]:

(1) $$\begin{array}{c} \%\text{ parastemia}=\frac{\text{total number of PRBC}}{\text{total number of RBC}}\times 100, \end{array}$$

where PRBC is parasitized red blood cells.

### 4.2. Red Blood Cells (RBC)

Average percentage of parasitemia suppression was calculated as follows:

(2) $$\begin{array}{c} \text{average \% of parastemia suppression}=\frac{\text{Av. \% of parasitemia in control}-\text{Av. \% of parasitemia in test}}{\text{Av. \% of parasitemia in control}}\times 100, \end{array}$$

where Av. = average.

### 4.3. Determination of Bodyweight

The bodyweights of the mice were measured to observe whether the test extract of *G. ternifolia* leaves prevented bodyweight loss. The weights were taken on day 0 (after an infection was initiated) and day 4 [25, 26]:

(3) $$\begin{array}{c} \text{body weight change}=\text{body weight day }4-\text{body weight day 0,}\\ \text{percent body weight change}=\frac{\text{body weight day }4-\text{body weight day 0}}{\text{body weight day }4}\times 100. \end{array}$$

### 4.4. Determination of Hemoglobin Level

The hemoglobin level of the mice was measured to evaluate the effectiveness of hydromethanolic crude extract and chloroform fraction *G. ternifolia* leaves prevented anemia. Hemoglobin was evaluated by using hemoglobin machine and hemoglobin cuvette. From vein section of tail of mice, blood sample was collected by a trained medical laboratory technologist with optimum pressure applied on the tail and filled microcuvette (Hb 301) approximately 10 *μ*l. Before inserting the microcuvette into the machine, excess blood was removed. The machine was run; hemoglobin level in g/dl was displayed automatically and recorded. Finally, mean hemoglobin level was determined [28]:

(4) $$\begin{array}{c} \text{mean hemoglobin}=\frac{\text{sum of hemoglobin for all mice in a group }\left(\text{in g/dl}\right)}{\text{total number of mice in that group}}. \end{array}$$

### 4.5. Determination of Mean Survival Time

Any death was recorded within 28 days of the study period to evaluate the hydromethanolic crude extract and chloroform fraction effect for improvement in survival days. The mice were maintained with free access to food and water [25, 26]:

(5) $$\begin{array}{c} \text{mean survival times}=\frac{\text{sum of survival times for all mice a group }\left(\text{in days}\right)}{\text{total number of mice in this group}}. \end{array}$$

### 4.6. Data Analysis

The results of the study were expressed as the mean ± standard error of the mean (*M* ± SEM). Comparison of parasitemia and statistical significance resolved by one-way ANOVA descriptive statistics. Post hoc tests for multiple comparisons of Tukey HSD and paired *t*-test were employed to test significance for the difference between initial and final results within the identical and in between groups using IBM SPSS for window (Version 20.0) statistical package. All data were analyzed at a 95% confidence interval and *P* < 0.05 was considered statistically significant.

## 5. Results

The yield and percent yield of hydromethanolic crude extract were found to be 42.6 g (10.65%) and stored in a refrigerator at 4°C until use.

### 5.1. Phytochemical Screening

The preliminary phytochemical screening of 80% hydromethanolic crude extract of *G. ternifolia* leaves for the probable presence of different secondary metabolites is summarized in Table 1.

### 5.2. Acute Toxicity Study

The acute toxicity study indicated that the hydromethanolic crude extract of *G. ternifolia* leaves did not cause mortality of mice within 24 hours up to 2000 mg/kg bodyweight. Gross physical and behavioral observation of the experimental mice also revealed no visible signs of acute toxicity like lacrimation, hair erection, and reduction in their motor and feeding activities. They were physically active for 14 days after plant extract administration.

### 5.3. Antimalarial Suppressive Test

Antimalarial suppressive test of hydromethanolic crude extract and chloroform fraction of *G. ternifolia* leaves in *P. berghei* infected mice resulted in reduced parasite load as compared to their respective negative control group. Positive control groups treated with chloroquine base 10 mg/kg/day cleared the parasite on day 4 under identical conditions. Hydromethanolic crude extract at 100 mg/kg/day, 200 mg/kg/day, and 400 mg/kg/day showed 27.09, 44.72, and 67.72% parasitemia inhibition, respectively, and chloroform fraction at 100 mg/kg/day, 200 mg/kg/day, and 400 mg/kg/day showed 35.21, 63.95, and 78.19% parasitemia inhibition, respectively, and the results were highly significant (*P* < 0.001) compared to the negative control group at day 4 (see Table 2).

**Figure 1:**
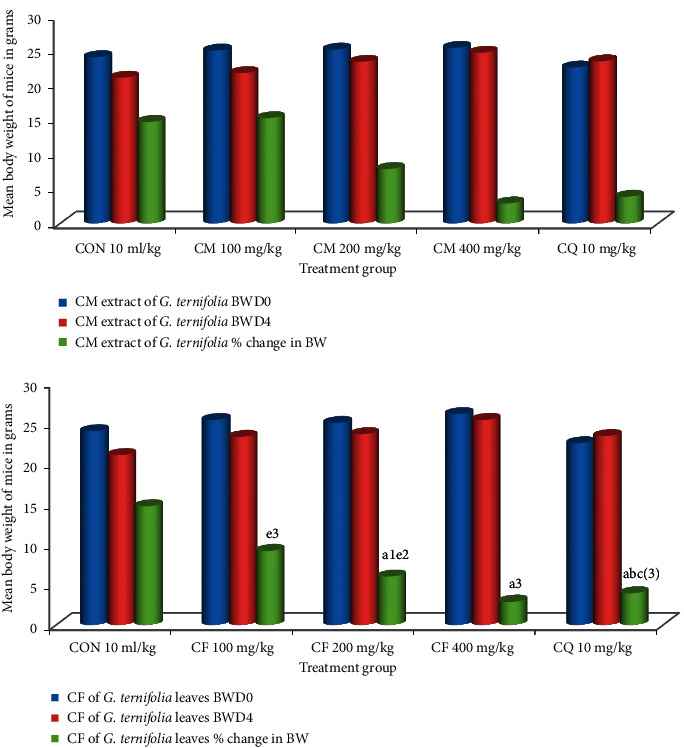
Effect of CM and CF of *G. ternifolia* on bodyweight of *P. berghei* infected mice in a 4-day suppressive test (N = 6, CON = negative control (solvent 10ml/kg), BWD0 = bodyweight at day zero, BWD4 = bodyweight at day 4, CM = crude methanolic extract, CF = chloroform fraction, CQ = chloroquine). a, compared to negative control; b, compared to 100 mg/kg; c, compared to 200 mg/kg; d, compared to 400 mg/kg; e, compared to CQ10 mg/kg: 1*P* < 0.05, 2*P* < 0.01, 3*P* < 0.001.

*Plasmodium berghei* infected mice treated with hydromethanolic crude extract of *G. ternifolia* leaves at 100 mg/kg/day −15.22 ± 1.88%(*P*=0.1000) change in bodyweight, 200 mg/kg/day −7.85 ± 1.65% (*P*=0.118) change in bodyweight, 400 mg/kg/day −2.90 ± 1.82% (*P* < 0.001) change in bodyweight and chloroform fraction at 100 mg/kg/day −9.14 ± 0.88% (*P*=0.324) change in bodyweight, 200 mg/kg/day −5.95 ± 0.72% (*P*=0.017) change in bodyweight, 400 mg/kg/day −2.85 ± 1.37% (*P* < 0.001) of bodyweight and chloroquine base at 10 mg/kg/day gain 3.87% (*P* < 0.001) of bodyweight and mice treated with solvent 10 ml/kg/day loss 14.68% of bodyweight (see Figure 1).

**Figure 2:**
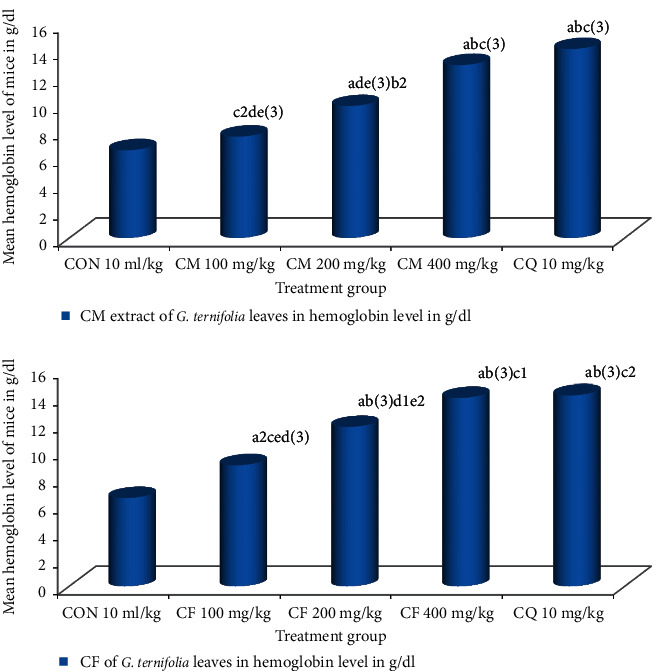
Effect of CM and CF of *G. ternifolia* on hemoglobin of *P. berghei* infected mice in a 4-day suppressive test (N = 6, CON = negative control (solvent 10 ml/kg), CM = crude hydromethanolic extract, CF = chloroform fraction, CQ = chloroquine). a, compared to negative control; b, compared to 100 mg/kg; c, compared to 200 mg/kg; d, compared to 400 mg/kg; e, compared to CQ10 mg/kg: 1*P* < 0.05, 2*P* < 0.01, 3*P* < 0.001.

The hydromethanolic crude extract of *G. ternifolia* leaves showed average hemoglobin levels at 100 mg/kg/day 7.57 ± 0.24 g/dl (*P*=0.668), 200 mg/kg/day 9.87 ± 0.40 g/dl (*P* < 0.001), 400 mg/kg/day 12.95 ± 0.54 g/dl (*P* < 0.001), chloroform fraction at 100 mg/kg/day 8.98 ± 0.32 g/dl (*P* < 0.001), 200 mg/kg/day 11.82 ± 0.13 g/dl (*P* < 0.001), 400 mg/kg/day 13.97 ± 0.40 g/dl (*P* < 0.001), chloroquine base at 10 mg/kg/day 14.15 ± 0.32 g/dl (*P* < 0.001) and non-infected mice 14.37 ± 0.24 g/dl (*P* < 0.001) compared to the negative control (6.55 ± 0.68 g/dl) (see Figure 2).

**Figure 3:**
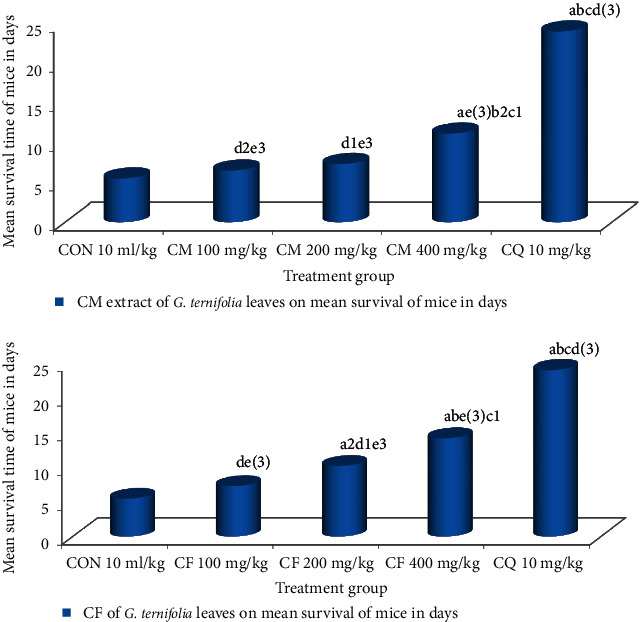
Effect of CM and CF of *G. ternifolia* on mean survival time of *P. berghei* infected mice in a 4-day suppressive test (N = 6, CON = negative control (solvent 10 ml/kg), CM = crude methanolic extract, CF = chloroform fraction, CQ = chloroquine). a, compared to negative control; b, compared to 100 mg/kg; c, compared to 200 mg/kg; d, compared to 400 mg/kg; e, compared to CQ 10 mg/kg: 1*P* < 0.05, 2*P* < 0.01, 3*P* < 0.001.

Hydromethanolic crude extract of *G. ternifolia* leaves at 100 mg/kg/day, 200 mg/kg/day, and 400 mg/kg/day showed 6.5 ± 0.22 (*P*=1.000), 7.330.33 (*P*=0.999), 11.17 ± 0.60 (*P*=0.440), chloroform fraction at 100 mg/kg/day, 200 mg/kg/day, 400 mg/kg/day 7.33 ± 0.33 (*P*=0.999), 10.17 ± 0.70 (*P*=0.689), 14.17 ± 0.31(*P*=0.042) and chloroquine base at 10 mg/kg/day showed 24.00 ± 1.83 (*P* < 0.001) days of mean survival time in four-day suppressive test, respectively, and chloroform fraction at 400 mg/kg/day showed significant difference (*P* < 0.05) in survival days compared to negative controls and infected mice treated with chloroquine base at 10 mg/kg/day showed highly significant (*P* < 0.001) in survival days compared to negative controls (5.50 ± 0.43) (see Figure 3).

### 5.4. Antimalarial Curative Test

Antimalarial curative test of hydromethanolic crude extract of *G. ternifolia* leaves at 100 mg/kg/day, 200 mg/kg/day, 400 mg/kg/day and chloroform fraction of *G. ternifolia leaves* at 100 mg/kg/day, 200 mg/kg/day, 400 mg/kg/day *and* chloroquine base 10 mg/kg/day in *P. berghei* infected mice at day 5 showed 25.58, 36.98, 48.76, 46.36, 62.87, 74.42, and 92.87 percent parasitemia inhibition, respectively (see Table 3).

**Figure 4:**
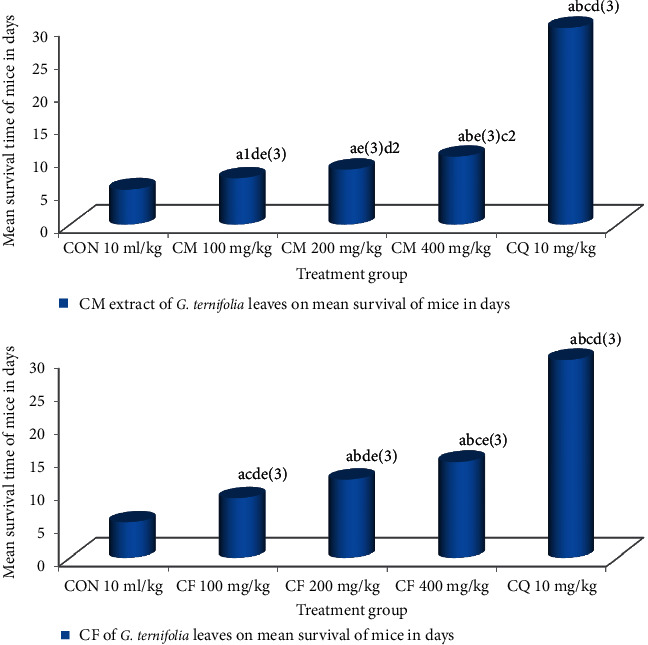
Effect of CM and CF of *G. ternifolia* on mean survival time of *P. berghei* infected mice in a curative test (N = 6, CON = negative control (solvent 10 ml/kg), CM = crude methanolic extract, CF = chloroform fraction, CQ = chloroquine). a, compared to negative control; b, compared to 100 mg/kg; c, compared to 200 mg/kg; d, compared to 400 mg/kg; e, compared to CQ 10 mg/kg: 1*P* < 0.05, 2*P* < 0.01, 3*P* < 0.001.

Hydromethanolic crude extract of *G. ternifolia* leaves at 100 mg/kg/day, 200 mg/kg/day, 400 mg/kg/day and chloroform fraction of *G. ternifolia* leaves at 100 mg/kg/day, 200 mg/kg/day, 400 mg/kg/day and chloroquine base at 10 mg/kg/day showed 7.00 ± 0.26 days (*p*=0.037), 8.33 ± 0.33 days, 10.33 ± 0.33 days, 9.00 ± 0.26 days, 11.83 ± 0.48 days, 14.50 ± 0.62 days and 30.00 ± 0.00 days of mean survival time in curative test, respectively, and were highly significant differences (*p* < 0.001) compared to negative control which was 5.33 ± 0.21 days (see Figure 4).

## 6. Discussion

Currently, malaria management causes an unlimited threat due to resistance of plasmodium species to available drugs [2, 3]. There is a requirement to possess a replacement and effective agent. Therefore, we assessed antimalarial activity of hydromethanolic crude extract and chloroform fraction of *G. ternifolia* leaves in *P. berghei* infected mice. Ethnomedical information indicates that *G. ternifolia* (Rubiaceae) has been employed by traditional healers to palliate malaria and its related fevers [6, 7]. It is also used to manage hemorrhoids lesions, diabetes, constipation, stomachache, and vermifuge activity [9], from the promising results of the in vitro cell culture of chloroquine-resistant Indochina (W2) and chloroquine sensitive to Sierra-Leone (D6) strains of *Plasmodium falciparum* [9]. The in vivo activities of hydromethanolic crude extract and chloroform fraction of *G. ternifolia* leaves were evaluated. The hydromethanolic crude extract yield of *G. ternifolia* leaves was 10.65% w/w which is not up to the *G. ternifolia* root bark (19% w/w) [12]. The medicinal plant (*G. ternifolia*) contains considerable secondary bioactive constituents like alkaloids, anthraquinones, flavonoids, phenols, saponins, sterols, tannins, and terpenoids [14–21]. The antimalarial effects of both hydromethanolic crude extract and chloroform fraction of *G. ternifolia* leaves have higher mean percent parasitemia inhibition, reduction in weight, prevention of anemia, and a rise in mean survival time in days in a dose-dependent manner. Alkaloids are attributed to their ability to intercalate with DNA and terminate division of cells [14]. Flavonoids have an ability to complex with extracellular and soluble proteins and to complex with organisms' cell components. More lipophilic flavonoids may disrupt microbial membranes, inactivate toxins, and inhibit isolated enzymes and complexing activities [10, 16]. Phenolic toxicity produced by phenolic compounds to microorganisms includes enzyme inhibition by the oxidized compounds, possibly through reaction with sulfhydryl groups or through more nonspecific interactions with the proteins [29]. Tannins stimulate phagocytic cells, host-mediated tumor activity complexation with proteins through so-called nonspecific forces like hydrogen bonding and hydrophobic effects, further by attractive force formation [30]. The terpenes' mechanism of action is not fully understood but is purported to involve membrane disruption by the lipophilic compounds [10, 16]. Hydroethanolic crude extract of *G. ternifolia* leaves did not cause any overt morbidity and mortality in the experimental mice at 2,000 mg/kg oral administration. This showed that a median lethal dose of 50% (LD_50_) is larger than 2,000 mg/kg, which attests that plant products are frequently considered to be safe and have fewer adverse effects than synthetic ones [22, 23]. However, species variations would limit such a clear-cut extrapolation of the findings of this study to humans. The in vivo antiplasmodial activities of hydromethanolic crude extract and chloroform fraction of *G. ternifolia* leaves were investigated by evaluating the chemosuppression during early infection and curative tests. Then, determination of percent parasitemia inhibition and improved survival days are widely used parameters for evaluating of antiplasmodial activities [25, 26]. Chloroform fraction at 100 mg/kg/day, 200 mg/kg/day, and 400 mg/kg/day showed higher percent parasitemia inhibition than the hydromethanolic crude extract [25, 26]. In the 4-day chemosuppressive and curative test, hydromethanolic crude extract and chloroform fraction of *G. ternifolia* leaves produced dose-dependent mean percent parasitemia inhibition and prolonged the survival days in *P. berghei* infected mice, the results recorded at the doses administered. Parasitemia level *P. berghei* infected mice treated with hydromethanolic crude extract and chloroform fraction of *G. ternifolia* leaves was due to a suppressive effect on the multiplication and erythrocyte infectivity of *P. berghei* parasites in mice. Chloroform fraction demonstrated dose-dependent higher activity in blood parasitemia inhibition in *P. berghei* than hydromethanolic crude extract. The relatively higher activity of the chloroform fraction might flow from/to secondary metabolites which can be partitioned during fractionation [9, 14–16]. The mean survival time may well be a significant parameter to determine the antimalarial activity of plant extract [25]. The hydromethanolic crude extract and chloroform fraction of *G. ternifolia* leaves prolonged the survival time of *P. berghei* infected mice dose dependently. This could be due to the presence of secondary metabolites that prevent the pathologic effect of the parasite in the infected mice [10, 14–16, 29].

Comparing the mean survival time of chloroform fraction and hydromethanolic crude extract, mice treated with chloroform fraction showed higher mean survival time; this may be due to the feed intake depressing effect of metabolites and interfere with the protein digestion by secondary metabolites that may be absent in chloroform fraction [31]. In comparing the result obtained from this study, the three doses of hydromethanolic crude extract and chloroform fraction of *G. ternifolia* leaves showed significant mean percent parasitemia inhibition compared to the negative control. In vivo antiplasmodial activity is additionally classified as moderate, good, and extremely good if extract displayed percent parasite inhibition is ≥50% at a dose of 500 mg/kg/day, 250 mg/kg/day, and 100 mg/kg/day in a 4-day suppressive test, respectively [32]. Supporting this classification, the hydromethanolic crude extract of *G. ternifolia* leaves at 400 mg/kg/day showed 67.72%, and chloroform fraction at 400 and 200 mg/kg/day showed 78.19% and 63.95% parasitemia inhibition in *P. berghei* infected mice, respectively, and exhibited moderate and good antiplasmodial activity. A stronger percentage of parasitemia inhibition was observed in chloroform fraction than the hydromethanolic crude extract at 100 mg/kg/day, 200 mg/kg/day, and 400 mg/kg/day. These results indicated that compounds which have antimalarial activity were soluble in the solvent accustomed extract and fractionate the leaves of *G. ternifolia* for antimalarial activity. Weight is eminence of plasmodium infection in mice, indicating that a decent antimalarial agent should prevent weight reduction in *Plasmodium* infected mice. The dose-dependent suppressive activities of hydromethanolic crude extract and chloroform fraction *G. ternifolia* leaves propose that hydromethanolic crude extract and chloroform fraction have not got any toxic effects in experimental mice at the doses evaluated. In the curative test, the mean percent parasitemia was increased in the negative control from the 3^rd^ day to the 5^th^ day. In the *Plasmodium berghei* infected mice treated with hydromethanolic crude extract and chloroform fraction of *G. ternifolia* leaves, the mean percent parasitemia increased from day 3 to day 4 and showed a decrease in percent parasitemia from the day 5 to day 7 in a dose-dependent manner compared to the negative control. The infected mice treated with chloroquine base showed a decrease in average percent parasitemia from 3^rd^ day to the 7^th^ day.

In the curative test, hydromethanolic crude extract and chloroform fraction of *G. ternifolia* leaves produced significant difference in percent hemoglobin level compared to the negative control. The hydromethanolic crude extract and chloroform fraction of *G. ternifolia* leaves at doses of 100, 200, and 400 mg/kg/day showed increased mean survival time with significant difference compared to the negative control and produced highly significant difference (*P* < 0.001) at 400 mg/kg/day compared to negative control. The promising result from the curative test suggests that the crude extract incorporates a therapeutic efficacy against established malarial parasitic infections.

## 7. Conclusion

The hydromethanolic crude extract and chloroform fraction of *G. ternifolia* leaves have in vivo anti-malarial activities. Chloroform fraction showed relatively better antimalarial effect in all parameters determined than hydromethanolic crude extract of *G. ternifolia* leaves. The dearth of acute toxicity and antimalarial activity upholds the earlier in vitro findings as well as the folkloric use in Ethiopian traditional medicine. However, species variation would also limit such a straightforward extrapolation of the findings of this study to humans.

### 7.1. Recommendation

Our study confirmed the claim in Ethiopian traditional medicine that the plant has therapeutic values in malarial infections. So, it needs further in-depth investigation by using primate and other models against *P. falciparum* infection are justifiable. *Gardenia ternifolia* is multipurpose which is employed for the building of homes, firewood, and animal feed and for treatment of various diseases in both humans and stock that the concerned bodies are advised to require measures to conserve it.

## Figures and Tables

**Table 1:** Phytoconstituents of hydromethanolic crude extract of *G. ternifolia* leaves.

Phytochemical	Type of test	Appearance when positive	Result
Alkaloids	Wagner's and Mayer's tests	Reddish brown and white creamy ppt	+
Anthraquinones	(Borntrager's test) chloroform + NaOH	Red colour	+
cardiac glycosides	(Keller–Killani test) glacial acetic acid + ferric chloride + concentrated sulfuric acid	A brown ring	-
Flavonoids	Alkaline reagent test	Colourless	+
Phenols	Ferric chloride	Bluish black	+
Resins	Ethanol + distilled water	Formation of a precipitate	-
Saponins	Foam test	Foam	+
Sterols	(Liebermann–Burchard test) chloroform + acetic acid + sulphuric acid	Blue green ring	+
Tannins	Ferric chloride	A brownish green or blue black coloration	+
Terpenoids	(Salkowski test) sulphuric acid + chloroform	Reddish brown	+

(+) indicates presence; (−) indicates absence; ppt.: precipitate.

**Table 2:** Mean percent parasitemia and mean percent parasitemia inhibition of *P. berghei-*infected mice treated with hydromethanolic crude extract and chloroform fraction of *Gardenia ternifolia* leaves in the 4-day suppressive test.

Extract	Mean percent parasitemia	Mean percent parasitemia inhibition
CON 10 ml/kg	43.00 ± 1.58	0.00
CM 100 mg/kg	31.35 ± 0.57acde (3)	27.09
CM 200 mg/kg	23.77 ± 0.52abde (3)	44.72
CM 400 mg/kg	13.88 ± 0.35abce (3)	67.72
CF 100 mg/kg	27.86 ± 0.67acde (3)	35.21
CF 200 mg/kg	15.50 ± 0.41abde (3)	63.95
CF 400 mg/kg	9.38 ± 0.25abce (3)	78.19
CQ 10 mg/kg	0.00 ± 0.00abcd (3)	100

Data are expressed as mean ± SEM; (n = 6, CON = control, CM, crude hydromethanolic extract, CF = chloroform fraction of *Gardenia ternifolia*, and CQ = chloroquine). a, compared to negative control; b, compared to 100 mg/kg; c, compared to 200 mg/kg; d, compared to 400 mg/kg; e, CQ10 mg/kg: 3*P* < 0.001.

**Table 3:** Mean percent parasitaemia and mean percent parasitemia inhibition of *P. berghei-*infected mice treated with hydromethanolic crude extract and chloroform fraction of *G. ternifolia* leaves in the Rane's test.

Extract	Day 3 % parasitemia	Day-4 % parasitemia	Day 5		Day 6 % parasitemia	Day 7 % parasitemia
			% parasitemia	% para. inhibition		
CON 10 ml/kg	14.10 ± 0.54	35.00 ± 0.56	43.00 ± 1.58	0	0	0
CM 100 mg/kg	15.00 ± 0.37	34.00 ± 0.83	32.00 ± 0.45	25.58acde (3)	25.03 ± 0.59	21.00 ± 0.48
CM 200 mg/kg	14.03 ± 0.60	27.03 ± 0.81	27.10 ± 0.40	36.98 abe (3)	21.00 ± 0.46	17.05 ± 0.34
CM 400 mg/kg	15.10 ± 0.18	26.07 ± 0.46	22.03 ± 0.22	48.76 abe (3)	19.00 ± 0.60	14.03 ± 0.23
CF 100 mg/kg	12.13 ± 0.20	29.00 ± 0.30	23.07 ± 0.33	46.36 ade (3) c1	21.00 ± 0.37	16.90 ± 0.23
CF 200 mg/kg	14.05 ± 0.31	25.00 ± 0.24	15.97 ± 0.32	62.87 ade (3) b1	11.10 ± 0.16	10.03 ± 0.35
CF 400 mg/kg	15.13 ± 0.53	20.00 ± 0.52	11.00 ± 0.47	74.42 abce (3)	9.00 ± 0.16	7.17 ± 0.26
CQ 10 mg/kg	13.10 ± 0.38	7.07 ± 0.14	3.07 ± 0.22	92.87 abcd (3)	0.00 ± 0.00	0.00 ± 0.00

Data are expressed as mean ± SEM; (n = 6: CQ = chloroquine, CON = control, CM, crude hydromethanolic extract, CF = chloroform fraction of *Gardenia ternifolia*, % par supp = % parasitemia suppression). a, compared to negative control; b, compared to100 mg/kg; c, compared to 200 mg/kg; d, compared to 400 mg/kg; e, compared to CQ10 mg/kg: 1*P* < 0.05, 3*P* < 0.001.

## Data Availability

All data generated or analyzed during this study have been attached in the supplementary files.

## Abbreviations

CQ: Chloroquine
EPHI: Ethiopian Public Health Institute
IC_50_: 50% inhibitory concentration
LD_50_: Median lethal dose 50
PRBCs: Parasitized red blood cells.

## Supplementary Materials

Supplementary Materials (1) Raw data: in vivo antimalarial effect of *Gardenia ternifolia* chemosuppressive data. (2) List of supplies, chemicals, reagents, and equipment used in our research.

## References

[1] World Health Organization (2020). *World Malaria Report 2020: 20 Years of Global Progress and Challenges*.

[2] East African Network for Monitoring Antimalarial Treatment (EANMAT) (2003). The efficacy of antimalarial monotherapies, sulphadoxine–pyrimethamine and amodiaquine in East Africa: implications for sub‐regional policy. *Tropical Medicine & International Health*.

[3] Talisuna A. O., Bloland P., D’Alessandro U. (2004). History, dynamics, and public health importance of malaria parasite resistance. *Clinical Microbiology Reviews*.

[4] Huxley A., Griffiths M., Levy M. (1992). *Dictionary of Gardening: The New Royal Horticultural Society*.

[5] von Maydell H. J. (2015). Trees and Shrubs of the Sahel-their characteristics and Uses. *Open Journal of Forestry*.

[6] Kokwaro J. O. (1993). *Medicinal Plants of East Africa*.

[7] Weenen H., Nkunya M., Bray D., Mwasumbi L., Kinabo L., Kilimali V. (1990). Antimalarial activity of Tanzanian medicinal plants. *Planta Medica*.

[8] Midiwo J. O., Matasi J. J., Wanjau O. M., Mwangi R. W., Waterman P. G., Wollenweber E. (1990). Anti-feedant effects of surface accumulated flavonoids of polygonum senegalense. *Bulletin of the Chemical Society of Ethiopia*.

[9] Achigan-Dako E. G., N’Danikou S., Assogba-Komlan F., Ambrose-Oji B., Ahanchede A., Pasquini M. W. (2011). Diversity, geographical, and consumption patterns of traditional vegetables in sociolinguistic communities in Benin: implications for domestication and utilization. *Economic Botany*.

[10] Ochieng C. O., Ogweno Mid J., Okinda Owu P. (2010). Anti-plasmodial and larvicidal effects of surface exudates ofGardenia ternifolia aerial parts. *Research Journal of Pharmacology*.

[11] Al-Adhroey A. H., Nor Z. M., Al-Mekhlafi H. M., Amran A. A., Mahmud R. (2011). Antimalarial activity of methanolic leaf extract of Piper betle L. *Molecules*.

[12] Nureye D., Assefa S., Nedi T., Engidawork E. (2018). In vivo antimalarial activity of the 80 Methanolic root bark extract and solvent fractions of Gardenia ternifolia Schumach. & Thonn. (Rubiaceae) against Plasmodium berghei. *Evidence-based complementary and alternative medicine*.

[13] Zohra S. F., Meriem B., Samira S., Muneer M. A. (2012). Phytochemical screening and identification of some compounds from mallow. *Journal of Natural Product and Plant Resources*.

[14] Alafid F., Edrah S. M., Meelad F. M., Belhaj S., Altwair K., Maizah N. R. (2019). Evaluation of phytochemical constituents and antibacterial activity of thymelaea hirsuta (l.) Endl, and that utilised as a conventional treatment of infertility and diabetic in Libya. *World Journal of Pharmaceutical Research*.

[15] Scalbert A. (1991). Antimicrobial properties of tannins. *Phytochemistry*.

[16] Usman H., Abdulrahman F. I., Usman A. (2009). Qualitative phytochemical screening and in vitro antimicrobial effects of methanol stem bark extract of *Ficus thonningii* (Moraceae). *African Journal of Traditional, Complementary and Alternative*.

[17] Cowan M. M. (1999). Plant products as antimicrobial agents. *Clinical Microbiology Reviews*.

[18] Dibua Uju M.-E., Okeke C. C., Chioma U., Chimaobi K. F., Austin O. (2013). *In vivo* antimalarial and cytotoxicity activity of ethanolic stem bark of *Petersianthus macrocarpus* and leaf of *Astonia boonei* in experimental mice model. *International Journal of Current Microbiology and Applied*.

[19] Othman L., Sleiman A., Abdel-Massih R. M. (2019). Antimicrobial activity of polyphenols and alkaloids in Middle eastern plants. *Frontiers in Microbiology*.

[20] Omojate Godstime C., Enwa Felix O., Jewo Augustina O., Eze Christopher O. (2014). Mechanisms of antimicrobial actions of phytochemicals against enteric pathogens–a review. *Journal of Pharmaceutical, Chemical and Biological Sciences*.

[21] Gollapudi S., Sharma H. A., Aggarwal S., Byers L. D., Ensley H. E., Gupta S. (1995). Isolation of a previously unidentified polysaccharide (MAR-10) from *Hyssop officinalis* that exhibits strong activity against human immunodeficiency virus type 1. *Biochemical and Biophysical Research Communications*.

[22] Organization for Economic Co-operation and Development (2008). *Test No. 425: Acute Oral Toxicity: Up-And-Down Procedure*.

[23] Mohr B. J., Fakoya F. A., Hau J., Souilem O., Anestidou L. (2016). The governance of animal care and use for scientific purposes in Africa and the Middle East. *ILAR Journal*.

[24] Parasuraman S. (2011). Toxicological screening. *Journal of Pharmacology and Pharmacotherapeutics*.

[25] Fidock D. A., Rosenthal P. J., Croft S. L., Brun R., Nwaka S. (2004). Antimalarial drug discovery: efficacy models for compound screening. Supplementary documents. *Trends Parasitol*.

[26] Bantie L., Assefa S., Teklehaimanot T., Engidawork E. (2014). *In vivo* antimalarial activity of the crude leaf extract and solvent fractions of *Croton macrostachyus* Hocsht. (Euphorbiaceae) against *Plasmodium berghei* in mice. *BMC Complementary and Alternative Medicine*.

[27] Iyiola O. A., Tijani A. Y., Lateef K. M. (2011). Antimalarial activity of ethanolic stem bark extract of *Alstonia boonei* in mice. *Asian Journal of Biological Sciences*.

[28] Rappaport A. I., Karakochuk C. D., Whitfield K. C., Kheang K. M., Green T. J. (2017). A method comparison study between two hemoglobinometer models (Hemocue Hb 301 and Hb 201+) to measure hemoglobin concentrations and estimate anemia prevalence among women in Preah Vihear, Cambodia. *International Journal of Laboratory Hematology*.

[29] Ozcan T., Akpinar-Bayizit A., Yilmaz-Ersan L., Delikanli B. (2014). Phenolics in human health. *International Journal of Chemical Engineering and Applications*.

[30] Gurney P. C., Elliott S. S., Vom E., Spence S. J., Bagnato S., Watkins A. (2008). Inventors; leica biosystems melbourne pty ltd, assignee. Method and apparatus for tissue sample processing. *United States Patent Application US*.

[31] Ryley J. F., Peters W. (1970). The antimalarial activity of some quinolone esters. *Annals of Tropical Medicine & Parasitology*.

[32] Deressa W., Olana D., Chibsa S. (2004). Magnitude of malaria admissions and deaths at hospitals and health centers in Oromia, Ethiopia. *Ethiopian Medical Journal*.

