# Supplementary material for: Antimalarial Activities of Hydromethanolic Crude Extract and Chloroform Fraction of Gardenia ternifolia Leaves in Plasmodium berghei Infected Mice
Source: Evid Based Complement Alternat Med. 2020 Dec 29;2020:6674002. doi: 10.1155/2020/6674002 (PMC7785367; doi:10.1155/2020/6674002)
Supplement: Supplementary Materials — (1) Raw data: in vivo antimalarial effect of Gardenia ternifolia chemosuppressive data. (2) List of supplies, chemicals, reagents, and equipment used in our research. [file 6674002.f1.zip › 6674002.f1/List of supplies.docx]

### List of supplies, Chemicals, reagents and Equipment used in our research

Distilled water, Chloroquine phosphate, Absolute methanol, Ethyl alcohol absolute 99.8& Chloroform, N- hexane, Carbon tetrachloride, petroleum ether, Glacial acetic acid, Sodium chloride 0.9% IV infusion, 3.8%Tri sodium citrate, Giemsa stain, emersion oil, Whatman filter paper 18cm diameter and 0.1μm pore diameter, microscopic slide frosted (1mm-1.2mm thick and 25.4mm x 76.2mm, beakers, funnels, Aluminum foil, measuring cylinder, Disposable glove (medium size), heparinized syringe with needle of 5/10ml, diabetic syringe, HemoCue machine, haemocue cuvette, Gauze 40 mesh, mice cage, mice gavage, dry oven 250 +10% Volts AC 600watts 50/60Hz, Electrical balance, pasture pipette, stainless scissor 17cm long and light microscope.
