# Supplementary material for: Antimalarial Activities of Hydromethanolic Crude Extract and Chloroform Fraction of Gardenia ternifolia Leaves in Plasmodium berghei Infected Mice
Source: Evid Based Complement Alternat Med. 2020 Dec 29;2020:6674002. doi: 10.1155/2020/6674002 (PMC7785367; doi:10.1155/2020/6674002)
Supplement: Supplementary Materials — (1) Raw data: in vivo antimalarial effect of Gardenia ternifolia chemosuppressive data. (2) List of supplies, chemicals, reagents, and equipment used in our research. [file 6674002.f1.zip › 6674002.f1/Raw data.docx]

**Raw data**

***In Vivo* antimalarial effect of *Gardenia ternifolia* chemosuppressive data**

|  | sub group | % para-1 | % para-2 | % para-3 | % para-4 | % para-5 | Av. % Para | BW Before | BW after | Bw diff | HGB g/dl | Suvi Date |  |
| --- | --- | --- | --- | --- | --- | --- | --- | --- | --- | --- | --- | --- | --- |
| CON | Neg 1- 1 | 42 | 40 | 46 | 48 | 45 | 44.2 | 21.2 | 17.1 | -4.1 | 5.9 | 6 | |
| CON | Neg 1 - 2 | 34 | 36 | 38 | 47 | 37 | 38.4 | 21.5 | 18.1 | -3.4 | 5.4 | 5 | |
| CON | Neg 1 - 3 | 47 | 45 | 48 | 46 | 56 | 48.4 | 24 | 21.2 | -2.8 | 5.4 | 4 | |
| CON | Neg 1 - 4 | 40 | 45 | 44 | 32 | 32 | 38.6 | 25.3 | 23.1 | -2.2 | 6.4 | 6 | |
| CON | Neg 1- 5 | 47 | 44 | 44 | 45 | 45 | 45 | 24 | 23.2 | -0.8 | 6.4 | 7 | |
| CON | Neg 1 - 6 | 40 | 45 | 43 | 40 | 49 | 43.4 | 28 | 23.5 | -4.5 | 9.8 | 5 | |
| CM100mg | CM100 2 - 1 | 36 | 32 | 29 | 33 | 37 | 33.4 | 22.4 | 20 | -2.4 | 7.8 | 6 | |
| CM100mg | CM100 2 - 2 | 27 | 32 | 29 | 31 | 28 | 29.4 | 25.2 | 22.3 | -2.9 | 7.3 | 6 | |
| CM100mg | CM100 2 - 3 | 27 | 28 | 34 | 32 | 31 | 30.4 | 26 | 23.1 | -2.9 | 8.5 | 7 | |
| CM100mg | CM100 2 - 4 | 30 | 29 | 35 | 31 | 32 | 31.4 | 24 | 19.5 | -4.5 | 7.7 | 7 | |
| CM100mg | CM100 2 - 5 | 31.5 | 33 | 33 | 32.5 | 31 | 32.2 | 26 | 23.2 | -2.8 | 7.3 | 7 | |
| CM100mg | CM100 2 - 6 | 30 | 33.5 | 33 | 28 | 32 | 31.3 | 26.1 | 22 | -4.1 | 6.8 | 6 | |
| CM200mg | CM200 3 - 1 | 22 | 29 | 23 | 21 | 22 | 23.4 | 27 | 26.2 | -0.8 | 8.9 | 8 | |
| CM200mg | CM200 3 - 2 | 21 | 23 | 25 | 28 | 27 | 24.8 | 25 | 24 | -1 | 9.8 | 7 | |
| CM200mg | CM200 3 - 3 | 26 | 22 | 18 | 29 | 21 | 23.2 | 26 | 24.3 | -1.7 | 11.3 | 6 | |
| CM200mg | CM200 3- 4 | 21 | 31 | 22 | 30 | 23 | 25.4 | 24.5 | 21.8 | -2.7 | 8.8 | 8 | |
| CM200mg | CM200 3 - 5 | 22 | 19 | 21 | 21 | 26 | 21.8 | 25 | 23.2 | -1.8 | 10.6 | 8 | |
| CM200mg | CM200 3 - 6 | 27 | 24 | 21 | 23 | 25 | 24 | 23 | 20.4 | -2.6 | 9.8 | 7 | |
| CM400mg | CM400 4 - 1 | 15 | 13 | 17 | 14 | 13 | 14.4 | 26.4 | 26.2 | -0.2 | 11 | 12 | |
| CM400mg | CM400 4 - 2 | 14 | 11.5 | 15 | 16 | 14 | 14.1 | 27 | 26.4 | -0.6 | 15 | 10 | |
| CM400mg | CM400 4- 3 | 14 | 19 | 12 | 13 | 10 | 13.6 | 25.6 | 24.9 | -0.7 | 12.6 | 9 | |
| CM400mg | CM400 4- 4 | 17 | 12 | 16 | 16 | 12 | 14.6 | 22 | 21.4 | -0.6 | 13 | 12 | |
| CM400mg | CM400 4 - 5 | 13 | 13 | 15 | 11 | 9.5 | 12.3 | 26 | 23.4 | -2.6 | 12.5 | 11 | |
| CM400mg | CM400 4 - 6 | 15 | 10.5 | 16 | 13 | 17 | 14.3 | 25 | 25.6 | 0.6 | 13.6 | 13 | |
| CF100mg | CF100 5- 1 | 31 | 37.5 | 25 | 27.5 | 30 | 30.2 | 24 | 22 | -2 | 9.7 | 8 | |
| CF100mg | CF100 5 - 2 | 27.5 | 22.5 | 25.5 | 31 | 26.5 | 26.6 | 22.5 | 20.3 | -2.2 | 10 | 7 | |
| CF100mg | CF100 5- 3 | 25.5 | 27 | 24 | 30.5 | 28 | 27 | 24.9 | 22.8 | -2.1 | 8.7 | 7 | |
| CF100mg | CF100 5- 4 | 29 | 27.5 | 27 | 28.5 | 31.5 | 28.7 | 27 | 25.1 | -1.9 | 9.2 | 8 | |
| CF100mg | CF100 5 - 5 | 26.5 | 28 | 26.5 | 28.5 | 28 | 27.5 | 26 | 23.2 | -2.8 | 8.3 | 6 | |
| CF100mg | CF100 5 - 6 | 27 | 24.5 | 25 | 24.5 | 27 | 25.6 | 28 | 26.4 | -1.6 | 8 | 8 | |
| CF200mg | CF200 6 - 1 | 16 | 19 | 16 | 16.5 | 15.5 | 16.6 | 26 | 24.7 | -1.3 | 11.6 | 13 | |
| CF200mg | CF200 6 - 2 | 17.5 | 15 | 19 | 15.5 | 16 | 16.6 | 24.8 | 23 | -1.8 | 12 | 10 | |
| CF200mg | CF200 6 - 3 | 17 | 13.5 | 16 | 19 | 13.5 | 15.8 | 25 | 24.3 | -0.7 | 11.6 | 11 | |
| CF200mg | CF200 6 - 4 | 16 | 14 | 14 | 12.5 | 14.5 | 14.2 | 26 | 24.3 | -1.7 | 12.1 | 9 | |
| CF200mg | CF200 6 - 5 | 16 | 14.5 | 17 | 12.5 | 13.5 | 14.7 | 23.4 | 22.1 | -1.3 | 12.2 | 8 | |
| CF200mg | CF200 6 - 6 | 18 | 13.5 | 15.5 | 14.5 | 14 | 15.1 | 25 | 23.4 | -1.6 | 11.4 | 10 | |
| CF400mg | CF200 7 - 1 | 14 | 8.5 | 11 | 9.5 | 8 | 10.2 | 26.5 | 26.8 | 0.3 | 14 | 14 | |
| CF400mg | CF400 7- 2 | 10.5 | 8.5 | 9.5 | 8.5 | 13 | 10 | 26 | 25.1 | -0.9 | 14.5 | 13 | |
| CF400mg | CF400 7- 3 | 9.5 | 7.5 | 9 | 8 | 9.5 | 8.7 | 25.6 | 25 | -0.6 | 13.5 | 14 | |
| CF400mg | CF400 7- 4 | 11.5 | 8.5 | 6.5 | 9 | 10.5 | 9.2 | 25.7 | 24.9 | -0.8 | 15.6 | 15 | |
| CF400mg | CF400 7 - 5 | 9 | 8 | 9 | 8.5 | 9.5 | 8.8 | 26.5 | 26.4 | -0.1 | 13.4 | 14 | |
| CF400mg | CF400 7 - 6 | 10.5 | 8 | 8.5 | 10.5 | 9.5 | 9.4 | 26.4 | 24.3 | -2.1 | 12.8 | 15 | |
| CHLO10mg | CQ10 8 - 1 | 0 | 0 | 0 | 0 | 0 | 0 | 21 | 22.5 | 1.5 | 13.5 | 25 | |
| CHLO10mg | CQ10 8 - 2 | 0 | 0 | 0 | 0 | 0 | 0 | 20 | 21 | 1 | 14.1 | 23 | |
| CHLO10mg | CQ10 8 - 3 | 0 | 0 | 0 | 0 | 0 | 0 | 24 | 25.2 | 1.2 | 14.5 | 24 | |
| CHLO10mg | CQ10 8 - 4 | 0 | 0 | 0 | 0 | 0 | 0 | 24 | 23.6 | -0.4 | 15 | 16 | |
| CHLO10mg | CQ10 8 - 5 | 0 | 0 | 0 | 0 | 0 | 0 | 23 | 23.9 | 0.9 | 14.8 | 27 | |
| CHLO10mg | CQ10 8 - 6 | 0 | 0 | 0 | 0 | 0 | 0 | 23 | 24.2 | 1.2 | 13 | 29 | |
| Non inf | Noninf 9 - 1 |  |  |  |  |  |  |  |  |  | 14.6 |  |  |
| Non inf | Noninf 9 - 2 |  |  |  |  |  |  |  |  |  | 13.5 |  |  |
| Non inf | Noninf 9 - 3 |  |  |  |  |  |  |  |  |  | 14.8 |  |  |
| Non inf | Noninf 9 - 4 |  |  |  |  |  |  |  |  |  | 14.5 |  |  |
| Non inf | Noninf 9 - 5 |  |  |  |  |  |  |  |  |  | 13.8 |  |  |
| Non inf | Noninf 9 - 6 |  |  |  |  |  |  |  |  |  | 15 |  |  |

Section 3 : *In vivo* antimalarial effect of *G. ternifolia* curative data

| Day 3 |  |  |  |  |  |  |  |
| --- | --- | --- | --- | --- | --- | --- | --- |
| Group | sub group | % para-1 | % para-2 | % para-3 | % para-4 | % para-5 | Av. % Para |
| 1 | CM100 1 - 1 | 15 | 15 | 13 | 16 | 15 | 14.8 |
| 1 | CM100 1 - 2 | 18 | 14 | 17 | 15 | 16 | 16 |
| 1 | CM100 1 - 3 | 13 | 16 | 14 | 15 | 14 | 14.4 |
| 1 | CM100 1 - 4 | 17 | 14 | 15 | 15 | 18 | 15.8 |
| 1 | CM100 1 - 5 | 13 | 16 | 12 | 13 | 14 | 13.6 |
| 1 | CM100 1 - 6 | 17 | 14 | 16 | 16 | 14 | 15.4 |
| 2 | CM200 2 - 1 | 14 | 13 | 14 | 12 | 12 | 13 |
| 2 | CM200 2 - 2 | 13 | 16 | 11 | 13 | 12 | 13 |
| 2 | CM200 2 - 3 | 13 | 12 | 13 | 12 | 15 | 13 |
| 2 | CM200 2- 4 | 14 | 19 | 22 | 16 | 12 | 16.6 |
| 2 | CM200 2 - 5 | 13 | 16 | 18 | 13 | 15 | 15 |
| 2 | CM200 2 - 6 | 12 | 13 | 14 | 16 | 13 | 13.6 |
| 3 | CM400 3 - 1 | 16 | 13 | 17 | 14 | 15 | 15 |
| 3 | CM400 3 - 2 | 14 | 16 | 13 | 12 | 18 | 14.6 |
| 3 | CM400 3- 3 | 15 | 14 | 16 | 17 | 16 | 15.6 |
| 3 | CM400 3- 4 | 12 | 16 | 13 | 17 | 18 | 15.2 |
| 3 | CM400 3 - 5 | 15 | 16 | 16 | 16 | 15 | 15.6 |
| 3 | CM400 3 - 6 | 16 | 17 | 12 | 13 | 15 | 14.6 |
| 4 | CF100 4- 1 | 12 | 13 | 12 | 14 | 11 | 12.4 |
| 4 | CF100 4 - 2 | 15 | 13 | 10 | 12 | 14 | 12.8 |
| 4 | CF100 4- 3 | 11 | 11 | 12 | 13 | 10 | 11.4 |
| 4 | CF100 4- 4 | 12 | 12 | 13 | 10 | 12 | 11.8 |
| 4 | CF100 4 - 5 | 10 | 15 | 12 | 11 | 14 | 12.4 |
| 4 | CF100 4 - 6 | 11 | 13 | 12 | 13 | 11 | 12 |
| 5 | CF200 5 - 1 | 13 | 13 | 16 | 16.5 | 15.5 | 14.8 |
| 5 | CF200 5 - 2 | 12 | 16 | 14 | 15.5 | 16 | 14.7 |
| 5 | CF200 5 - 3 | 16 | 10 | 11.5 | 13 | 13.5 | 12.8 |
| 5 | CF200 5 - 4 | 13 | 14 | 14 | 14 | 16 | 14.2 |
| 5 | CF200 5 - 5 | 12 | 13 | 17 | 12.5 | 13.5 | 13.6 |
| 5 | CF200 5 - 6 | 13 | 14 | 15.5 | 14.5 | 14 | 14.2 |
| 6 | CF400 6 - 1 | 13 | 12 | 14 | 11 | 16 | 13.2 |
| 6 | CF400 6- 2 | 14 | 12 | 18 | 14 | 16 | 14.8 |
| 6 | CF400 6- 3 | 15 | 13 | 18 | 14 | 16 | 15.2 |
| 6 | CF400 6- 4 | 13 | 12 | 16 | 14 | 18 | 14.6 |
| 6 | CF400 6 - 5 | 19 | 16 | 18 | 17 | 15 | 17 |
| 6 | CF400 6 - 6 | 15 | 17 | 13 | 16 | 19 | 16 |
| 7 | Neg 7 - 1 | 13 | 16 | 19 | 15 | 14 | 15.4 |
| 7 | Neg 7 - 2 | 12 | 13 | 17 | 14 | 15 | 14.2 |
| 7 | Neg 7 - 3 | 14 | 13 | 16 | 13 | 17 | 14.6 |
| 7 | Neg 7 - 4 | 10 | 10 | 12 | 11 | 15 | 11.6 |
| 7 | Neg 7 - 5 | 11 | 17 | 16 | 15 | 15 | 14.8 |
| 7 | Neg 7 - 6 | 12 | 15 | 16 | 13 | 14 | 14 |
| 8 | CQ10 8 - 1 | 12 | 13 | 12 | 14 | 11 | 12.4 |
| 8 | CQ10 8 - 2 | 15 | 13 | 16 | 12 | 15 | 14.2 |
| 8 | CQ10 8 - 3 | 14 | 11 | 15 | 13 | 16 | 13.8 |
| 8 | CQ10 8 - 4 | 16 | 12 | 15 | 14 | 12 | 13.8 |
| 8 | CQ10 8 - 5 | 14 | 11 | 10 | 11 | 14 | 12 |
| 8 | CQ10 8 - 6 | 11 | 15 | 12 | 13 | 11 | 12.4 |
| Day 4 |  |  |  |  |  |  |  |
| Group | sub group | % para-1 | % para-2 | % para-3 | % para-4 | % para-5 | Av. % Para |
| 1 | CM100 1 - 1 | 34 | 37 | 39 | 36 | 35 | 36.2 |
| 1 | CM100 1 - 2 | 34 | 36 | 33 | 32 | 35 | 34 |
| 1 | CM100 1 - 3 | 36 | 25 | 37 | 29 | 24 | 30.2 |
| 1 | CM100 1 - 4 | 30 | 36 | 32 | 38 | 35 | 34.2 |
| 1 | CM100 1 - 5 | 34 | 35 | 34 | 37 | 36 | 35.2 |
| 1 | CM100 1 - 6 | 32 | 31 | 34 | 35 | 39 | 34.2 |
| 2 | CM200 2 - 1 | 28 | 28 | 29 | 26 | 23 | 26.8 |
| 2 | CM200 2 - 2 | 31 | 29 | 28 | 32 | 27 | 29.4 |
| 2 | CM200 2 - 3 | 29 | 28 | 31 | 29 | 27 | 28.8 |
| 2 | CM200 2- 4 | 24 | 25 | 22 | 23 | 25 | 23.8 |
| 2 | CM200 2 - 5 | 27 | 31 | 26 | 28 | 23 | 27 |
| 2 | CM200 2 - 6 | 24 | 23 | 28 | 28 | 29 | 26.4 |
| 3 | CM400 3 - 1 | 25 | 26 | 27 | 24 | 27 | 25.8 |
| 3 | CM400 3 - 2 | 26 | 29 | 28 | 32 | 25 | 28 |
| 3 | CM400 3- 3 | 24 | 24 | 24 | 25 | 27 | 24.8 |
| 3 | CM400 3- 4 | 26 | 23 | 25 | 25 | 27 | 25.2 |
| 3 | CM400 3 - 5 | 28 | 25 | 24 | 26 | 28 | 26.2 |
| 3 | CM400 3 - 6 | 26 | 27 | 25 | 27 | 27 | 26.4 |
| 4 | CF100 4- 1 | 28 | 29 | 27 | 29 | 28 | 28.2 |
| 4 | CF100 4 - 2 | 26 | 27 | 28 | 32 | 28 | 28.2 |
| 4 | CF100 4- 3 | 27 | 29 | 31 | 32 | 31 | 30 |
| 4 | CF100 4- 4 | 28 | 27 | 29 | 32 | 28 | 28.8 |
| 4 | CF100 4 - 5 | 29 | 31 | 27 | 29 | 32 | 29.6 |
| 4 | CF100 4 - 6 | 28 | 29 | 29 | 31 | 29 | 29.2 |
| 5 | CF200 5 - 1 | 26 | 24 | 25 | 29 | 27 | 26.2 |
| 5 | CF200 5 - 2 | 28 | 23 | 26 | 24 | 23 | 24.8 |
| 5 | CF200 5 - 3 | 27 | 24 | 26 | 22 | 25 | 24.8 |
| 5 | CF200 5 - 4 | 22 | 22 | 27 | 25 | 27 | 24.6 |
| 5 | CF200 5 - 5 | 26 | 24 | 25 | 26 | 23 | 24.8 |
| 5 | CF200 5 - 6 | 24 | 25 | 26 | 24 | 25 | 24.8 |
| 6 | CF400 6 - 1 | 22 | 21 | 18 | 21 | 20 | 20.4 |
| 6 | CF400 6- 2 | 21 | 19 | 20 | 21 | 19 | 20 |
| 6 | CF400 6- 3 | 19 | 20 | 20 | 19 | 23 | 20.2 |
| 6 | CF400 6- 4 | 18 | 16 | 19 | 17 | 18 | 17.6 |
| 6 | CF400 6 - 5 | 21 | 23 | 21 | 20 | 22 | 21.4 |
| 6 | CF400 6 - 6 | 20 | 19 | 23 | 22 | 18 | 20.4 |
| 7 | Neg 7 - 1 | 32 | 38 | 36 | 38 | 35 | 35.8 |
| 7 | Neg 7 - 2 | 34 | 38 | 37 | 36 | 37 | 36.4 |
| 7 | Neg 7 - 3 | 36 | 35 | 38 | 37 | 36 | 36.4 |
| 7 | Neg 7 - 4 | 34 | 35 | 33 | 32 | 32 | 33.2 |
| 7 | Neg 7 - 5 | 37 | 34 | 34 | 36 | 29 | 34 |
| 7 | Neg 7 - 6 | 38 | 35 | 33 | 36 | 29 | 34.2 |
| 8 | CQ10 8 - 1 | 7 | 6 | 8 | 5 | 6 | 6.4 |
| 8 | CQ10 8 - 2 | 7 | 9 | 6 | 6 | 8 | 7.2 |
| 8 | CQ10 8 - 3 | 9 | 6 | 6 | 7 | 9 | 7.4 |
| 8 | CQ10 8 - 4 | 6 | 9 | 5 | 8 | 7 | 7 |
| 8 | CQ10 8 - 5 | 6 | 8 | 7 | 7 | 8 | 7.2 |
| 8 | CQ10 8 - 6 | 6 | 9 | 8 | 5 | 8 | 7.2 |
| Day 5 |  |  |  |  |  |  |  |
| Group | sub group | % para-1 | % para-2 | % para-3 | % para-4 | % para-5 | Av. % Para |
| 1 | CM100 1 - 1 | 33 | 32 | 29 | 33 | 34 | 32.2 |
| 1 | CM100 1 - 2 | 27 | 31 | 29 | 35 | 28 | 30 |
| 1 | CM100 1 - 3 | 32 | 28 | 32 | 32 | 34 | 31.6 |
| 1 | CM100 1 - 4 | 35 | 34 | 31 | 31 | 32 | 32.6 |
| 1 | CM100 1 - 5 | 32 | 34 | 32 | 33 | 31 | 32.4 |
| 1 | CM100 1 - 6 | 34 | 35 | 33 | 32 | 32 | 33.2 |
| 2 | CM200 2 - 1 | 26 | 28 | 27 | 28 | 29 | 27.6 |
| 2 | CM200 2 - 2 | 28 | 29 | 26 | 24 | 28 | 27 |
| 2 | CM200 2 - 3 | 29 | 23 | 26 | 25 | 24 | 25.4 |
| 2 | CM200 2- 4 | 25 | 31 | 29 | 26 | 24 | 27 |
| 2 | CM200 2 - 5 | 25 | 29 | 28 | 26 | 28 | 27.2 |
| 2 | CM200 2 - 6 | 29 | 32 | 28 | 26 | 27 | 28.4 |
| 3 | CM400 3 - 1 | 22 | 23 | 25 | 21 | 20 | 22.2 |
| 3 | CM400 3 - 2 | 20 | 22 | 20 | 23 | 21 | 21.2 |
| 3 | CM400 3- 3 | 22 | 23 | 22 | 23 | 23 | 22.6 |
| 3 | CM400 3- 4 | 22 | 22 | 20 | 23 | 22 | 21.8 |
| 3 | CM400 3 - 5 | 20 | 21 | 24 | 21 | 23 | 21.8 |
| 3 | CM400 3 - 6 | 21 | 23 | 22 | 24 | 23 | 22.6 |
| 4 | CF100 4- 1 | 21 | 23 | 22 | 23 | 20 | 21.8 |
| 4 | CF100 4 - 2 | 24 | 22 | 23 | 21 | 28 | 23.6 |
| 4 | CF100 4- 3 | 23 | 25 | 24 | 20 | 25 | 23.4 |
| 4 | CF100 4- 4 | 24 | 23 | 22 | 21 | 26 | 23.2 |
| 4 | CF100 4 - 5 | 21 | 24 | 26 | 25 | 24 | 24 |
| 4 | CF100 4 - 6 | 20 | 23 | 25 | 21 | 23 | 22.4 |
| 5 | CF200 5 - 1 | 16 | 19 | 16 | 16.5 | 15.5 | 16.6 |
| 5 | CF200 5 - 2 | 17.5 | 17 | 14 | 15.5 | 18 | 16.4 |
| 5 | CF200 5 - 3 | 17 | 13.5 | 17 | 18 | 17 | 16.5 |
| 5 | CF200 5 - 4 | 16 | 17 | 14 | 18 | 14.5 | 15.9 |
| 5 | CF200 5 - 5 | 16 | 14.5 | 15 | 19 | 15 | 15.9 |
| 5 | CF200 5 - 6 | 13 | 15 | 15.5 | 15 | 14 | 14.5 |
| 6 | CF400 6 - 1 | 9.5 | 14 | 10 | 13 | 12 | 11.7 |
| 6 | CF400 6- 2 | 10.5 | 8.5 | 9.5 | 8.5 | 13 | 10 |
| 6 | CF400 6- 3 | 10 | 13 | 9 | 10 | 9.5 | 10.3 |
| 6 | CF400 6- 4 | 11.5 | 13 | 13.5 | 12 | 13.5 | 12.7 |
| 6 | CF400 6 - 5 | 9 | 10 | 14 | 12.5 | 12 | 11.5 |
| 6 | CF400 6 - 6 | 10.5 | 10 | 8.5 | 10.5 | 9.5 | 9.8 |
| 7 | Neg 7 - 1 | 42 | 40 | 46 | 48 | 45 | 44.2 |
| 7 | Neg 7 - 2 | 34 | 36 | 38 | 47 | 37 | 38.4 |
| 7 | Neg 7 - 3 | 47 | 45 | 48 | 46 | 56 | 48.4 |
| 7 | Neg 7 - 4 | 40 | 45 | 44 | 32 | 32 | 38.6 |
| 7 | Neg 7 - 5 | 47 | 44 | 44 | 45 | 45 | 45 |
| 7 | Neg 7 - 6 | 40 | 45 | 43 | 40 | 49 | 43.4 |
| 8 | CQ10 8 - 1 | 3 | 5 | 4 | 3 | 2 | 3.4 |
| 8 | CQ10 8 - 2 | 4 | 2 | 3 | 4 | 2 | 3 |
| 8 | CQ10 8 - 3 | 2 | 3 | 2 | 0 | 4 | 2.2 |
| 8 | CQ10 8 - 4 | 3 | 4 | 2 | 3 | 4 | 3.2 |
| 8 | CQ10 8 - 5 | 3 | 2 | 4 | 3 | 2 | 2.8 |
| 8 | CQ10 8 - 6 | 3 | 4 | 3 | 4 | 5 | 3.8 |
| Day 6 |  |  |  |  |  |  |  |
| Group | sub group | % para | % para | % para | % para | % para | Av. % Para |
| 1 | CM100 1 - 1 | 26 | 23 | 29 | 22 | 24 | 24.8 |
| 1 | CM100 1 - 2 | 27 | 24 | 23 | 21 | 24 | 23.8 |
| 1 | CM100 1 - 3 | 27 | 25 | 28.5 | 28.5 | 23 | 26.4 |
| 1 | CM100 1 - 4 | 24 | 29 | 28.5 | 26 | 28.5 | 27.2 |
| 1 | CM100 1 - 5 | 24 | 25 | 26 | 22 | 23 | 24 |
| 1 | CM100 1 - 6 | 25 | 24 | 23 | 26 | 22 | 24 |
| 2 | CM200 2 - 1 | 21 | 19 | 22 | 21 | 23 | 21.2 |
| 2 | CM200 2 - 2 | 18 | 18.5 | 23 | 19.5 | 18 | 19.4 |
| 2 | CM200 2 - 3 | 24 | 22 | 26 | 20 | 19 | 22.2 |
| 2 | CM200 2- 4 | 21 | 19 | 22 | 21.5 | 16 | 19.9 |
| 2 | CM200 2 - 5 | 20 | 19 | 21 | 24 | 23 | 21.4 |
| 2 | CM200 2 - 6 | 19 | 21 | 24 | 23 | 22.5 | 21.9 |
| 3 | CM400 3 - 1 | 22 | 21 | 19 | 18 | 20 | 20 |
| 3 | CM400 3 - 2 | 20 | 18 | 19 | 22 | 19 | 19 |
| 3 | CM400 3- 3 | 21 | 20 | 18 | 19 | 16 | 18.8 |
| 3 | CM400 3- 4 | 15 | 16 | 18 | 15 | 17 | 16.2 |
| 3 | CM400 3 - 5 | 16 | 21 | 22 | 21 | 21 | 20.2 |
| 3 | CM400 3 - 6 | 20 | 22 | 20 | 16 | 21 | 19.8 |
| 4 | CF100 4- 1 | 21 | 18 | 21 | 19 | 20 | 19.8 |
| 4 | CF100 4 - 2 | 20 | 22 | 19 | 21 | 19 | 20.2 |
| 4 | CF100 4- 3 | 19 | 21 | 24 | 20 | 21 | 21 |
| 4 | CF100 4- 4 | 25 | 22 | 19 | 22 | 21 | 21.8 |
| 4 | CF100 4 - 5 | 21 | 22 | 24 | 23 | 21 | 22.2 |
| 4 | CF100 4 - 6 | 23 | 20 | 19 | 22 | 21 | 21 |
| 5 | CF200 5 - 1 | 12 | 10 | 11 | 12 | 12 | 11.4 |
| 5 | CF200 5 - 2 | 12 | 12 | 10 | 11 | 12 | 11.4 |
| 5 | CF200 5 - 3 | 10 | 10 | 12 | 13 | 10 | 11 |
| 5 | CF200 5 - 4 | 10 | 12 | 9 | 10 | 11 | 10.4 |
| 5 | CF200 5 - 5 | 10 | 12 | 12 | 12 | 11 | 11.4 |
| 5 | CF200 5 - 6 | 12 | 10 | 12 | 11 | 10 | 11 |
| 6 | CF400 6 - 1 | 7 | 8.5 | 9 | 10 | 8 | 8.5 |
| 6 | CF400 6- 2 | 10.5 | 8.5 | 9.5 | 8.5 | 7 | 8.8 |
| 6 | CF400 6- 3 | 9.5 | 7.5 | 9 | 8 | 9.5 | 8.7 |
| 6 | CF400 6- 4 | 11.5 | 8.5 | 6.5 | 9 | 10.5 | 9.2 |
| 6 | CF400 6 - 5 | 9 | 11 | 9 | 8.5 | 9.5 | 9.4 |
| 6 | CF400 6 - 6 | 10.5 | 8 | 8.5 | 10.5 | 9.5 | 9.4 |
| 7 | Neg 7 - 1 |  |  |  |  |  |  |
| 7 | Neg 7 - 2 |  |  |  |  |  |  |
| 7 | Neg 7 - 3 |  |  |  |  |  |  |
| 7 | Neg 7 - 4 |  |  |  |  |  |  |
| 7 | Neg 7 - 5 |  |  |  |  |  |  |
| 7 | Neg 7 - 6 |  |  |  |  |  |  |
| 8 | CQ10 8 - 1 | 0 | 0 | 0 | 0 | 0 | 0 |
| 8 | CQ10 8 - 2 | 0 | 0 | 0 | 0 | 0 | 0 |
| 8 | CQ10 8 - 3 | 0 | 0 | 0 | 0 | 0 | 0 |
| 8 | CQ10 8 - 4 | 0 | 0 | 0 | 0 | 0 | 0 |
| 8 | CQ10 8 - 5 | 0 | 0 | 0 | 0 | 0 | 0 |
| 8 | CQ10 8 - 6 | 0 | 0 | 0 | 0 | 0 | 0 |
| Day 7 |  |  |  |  |  |  |  |
| Group | sub group | % para | % para | % para | % para | % para | Av. % Para |
| 1 | CM100 1 - 1 | 22 | 22 | 19 | 23 | 24 | 22 |
| 1 | CM100 1 - 2 | 19 | 24 | 23 | 21 | 19 | 21.2 |
| 1 | CM100 1 - 3 | 17 | 23 | 19 | 18 | 19 | 19.2 |
| 1 | CM100 1 - 4 | 20 | 21 | 22 | 23 | 19 | 21 |
| 1 | CM100 1 - 5 | 21 | 18 | 20 | 22 | 20 | 20.2 |
| 1 | CM100 1 - 6 | 20 | 23 | 23 | 24 | 22 | 22.4 |
| 2 | CM200 2 - 1 | 16 | 17 | 17 | 18 | 17 | 17 |
| 2 | CM200 2 - 2 | 18 | 18.5 | 15.5 | 16 | 15 | 16.6 |
| 2 | CM200 2 - 3 | 19 | 16 | 18 | 16 | 16 | 17 |
| 2 | CM200 2- 4 | 16 | 19 | 22 | 19 | 16 | 18.4 |
| 2 | CM200 2 - 5 | 16 | 19 | 17 | 18 | 17 | 17.4 |
| 2 | CM200 2 - 6 | 15 | 17 | 15 | 15 | 17.5 | 15.9 |
| 3 | CM400 3 - 1 | 12 | 13 | 15 | 14 | 13 | 13.4 |
| 3 | CM400 3 - 2 | 16 | 14 | 15 | 13 | 14 | 14.4 |
| 3 | CM400 3- 3 | 13 | 12 | 16 | 13 | 17 | 14.2 |
| 3 | CM400 3- 4 | 12 | 12 | 16 | 13 | 14 | 13.4 |
| 3 | CM400 3 - 5 | 13 | 17 | 14 | 18 | 12 | 14.8 |
| 3 | CM400 3 - 6 | 15 | 13 | 12 | 13 | 17 | 14 |
| 4 | CF100 4- 1 | 15 | 17 | 21 | 18 | 16 | 17.4 |
| 4 | CF100 4 - 2 | 17 | 14 | 16 | 20 | 17 | 16.8 |
| 4 | CF100 4- 3 | 15 | 18 | 14 | 17 | 16 | 16 |
| 4 | CF100 4- 4 | 19 | 16 | 15 | 19 | 19 | 17.6 |
| 4 | CF100 4 - 5 | 16 | 19 | 17 | 15 | 17 | 16.8 |
| 4 | CF100 4 - 6 | 15 | 16 | 18 | 16 | 19 | 16.8 |
| 5 | CF200 5 - 1 | 11 | 9 | 10 | 8 | 9 | 9.4 |
| 5 | CF200 5 - 2 | 8 | 12 | 9 | 10 | 12 | 10.2 |
| 5 | CF200 5 - 3 | 11 | 10 | 7 | 9 | 9 | 9.2 |
| 5 | CF200 5 - 4 | 8 | 11 | 10 | 9 | 12 | 10 |
| 5 | CF200 5 - 5 | 11 | 9 | 10 | 8 | 11 | 9.8 |
| 5 | CF200 5 - 6 | 12 | 13 | 10 | 11 | 12 | 11.6 |
| 6 | CF400 6 - 1 | 6 | 7 | 6 | 7 | 8 | 6.8 |
| 6 | CF400 6- 2 | 8 | 5.5 | 6 | 6 | 7 | 6.5 |
| 6 | CF400 6- 3 | 7 | 8 | 5 | 8 | 6 | 6.8 |
| 6 | CF400 6- 4 | 7 | 7 | 6 | 9 | 6 | 7 |
| 6 | CF400 6 - 5 | 9 | 8 | 9 | 8 | 6.5 | 8.1 |
| 6 | CF400 6 - 6 | 7 | 8 | 8 | 9 | 7 | 7.8 |
| 7 | Neg 7 - 1 |  |  |  |  |  |  |
| 7 | Neg 7 - 2 |  |  |  |  |  |  |
| 7 | Neg 7 - 3 |  |  |  |  |  |  |
| 7 | Neg 7 - 4 |  |  |  |  |  |  |
| 7 | Neg 7 - 5 |  |  |  |  |  |  |
| 7 | Neg 7 - 6 |  |  |  |  |  |  |
| 8 | CQ10 8 - 1 | 0 | 0 | 0 | 0 | 0 | 0 |
| 8 | CQ10 8 - 2 | 0 | 0 | 0 | 0 | 0 | 0 |
| 8 | CQ10 8 - 3 | 0 | 0 | 0 | 0 | 0 | 0 |
| 8 | CQ10 8 - 4 | 0 | 0 | 0 | 0 | 0 | 0 |
| 8 | CQ10 8 - 5 | 0 | 0 | 0 | 0 | 0 | 0 |
| 8 | CQ10 8 - 6 | 0 | 0 | 0 | 0 | 0 | 0 |
|  |  |  |  |  |  |  |  |
| Group | sub group | SurDate |  |  |  |  |  |
| 1 | Neg 1- 1 | 5 |  |  |  |  |  |
| 1 | Neg 1 - 2 | 6 |  |  |  |  |  |
| 1 | Neg 1 - 3 | 6 |  |  |  |  |  |
| 1 | Neg 1 - 4 | 5 |  |  |  |  |  |
| 1 | Neg 1 - 5 | 5 |  |  |  |  |  |
| 1 | Neg 1 - 6 | 5 |  |  |  |  |  |
| 2 | CM100 2 - 1 | 7 |  |  |  |  |  |
| 2 | CM100 2 - 2 | 8 |  |  |  |  |  |
| 2 | CM100 2 - 3 | 7 |  |  |  |  |  |
| 2 | CM100 2 - 4 | 7 |  |  |  |  |  |
| 2 | CM100 2 - 5 | 6 |  |  |  |  |  |
| 2 | CM100 2 - 6 | 7 |  |  |  |  |  |
| 3 | CM200 3 - 1 | 7 |  |  |  |  |  |
| 3 | CM200 3 - 2 | 9 |  |  |  |  |  |
| 3 | CM200 3 - 3 | 9 |  |  |  |  |  |
| 3 | CM200 3- 4 | 9 |  |  |  |  |  |
| 3 | CM200 3 - 5 | 8 |  |  |  |  |  |
| 3 | CM200 3 - 6 | 8 |  |  |  |  |  |
| 4 | CM400 4 - 1 | 11 |  |  |  |  |  |
| 4 | CM400 4- 2 | 10 |  |  |  |  |  |
| 4 | CM400 4- 3 | 11 |  |  |  |  |  |
| 4 | CM400 4- 4 | 10 |  |  |  |  |  |
| 4 | CM400 4 - 5 | 9 |  |  |  |  |  |
| 4 | CM400 4 - 6 | 11 |  |  |  |  |  |
| 5 | CF100 5- 1 | 9 |  |  |  |  |  |
| 5 | CF100 5 - 2 | 9 |  |  |  |  |  |
| 5 | CF100 5- 3 | 8 |  |  |  |  |  |
| 5 | CF100 5- 4 | 9 |  |  |  |  |  |
| 5 | CF100 5 - 5 | 10 |  |  |  |  |  |
| 5 | CF100 5 - 6 | 9 |  |  |  |  |  |
| 6 | CF200 6 - 1 | 11 |  |  |  |  |  |
| 6 | CF200 6 - 2 | 13 |  |  |  |  |  |
| 6 | CF200 6 - 3 | 12 |  |  |  |  |  |
| 6 | CF200 6- 4 | 13 |  |  |  |  |  |
| 6 | CF200 6 - 5 | 10 |  |  |  |  |  |
| 6 | CF200 6 - 6 | 12 |  |  |  |  |  |
| 7 | CF400 7- 1 | 15 |  |  |  |  |  |
| 7 | CF400 7- 2 | 13 |  |  |  |  |  |
| 7 | CF400 7- 3 | 13 |  |  |  |  |  |
| 7 | CF400 7- 4 | 14 |  |  |  |  |  |
| 7 | CF400 7 - 5 | 15 |  |  |  |  |  |
| 7 | CF400 7- 6 | 17 |  |  |  |  |  |
| 8 | CQ10 8 - 1 | 30 |  |  |  |  |  |
| 8 | CQ10 8 - 2 | 30 |  |  |  |  |  |
| 8 | CQ10 8 - 3 | 30 |  |  |  |  |  |
| 8 | CQ10 8 - 4 | 30 |  |  |  |  |  |
| 8 | CQ10 8 - 5 | 30 |  |  |  |  |  |
| 8 | CQ10 8 - 6 | 30 |  |  |  |  |  |
